# Acute Nonspecific Mesenteric Lymphadenitis: More Than “No Need for Surgery”

**DOI:** 10.1155/2017/9784565

**Published:** 2017-02-02

**Authors:** Rossana Helbling, Elisa Conficconi, Marina Wyttenbach, Cecilia Benetti, Giacomo D. Simonetti, Mario G. Bianchetti, Flurim Hamitaga, Sebastiano A. G. Lava, Emilio F. Fossali, Gregorio P. Milani

**Affiliations:** ^1^Pediatric Department of Southern Switzerland, Bellinzona, Switzerland; ^2^Department of Radiology, Ospedale Regionale Bellinzona e Valli, Bellinzona, Switzerland; ^3^University Children's Hospital Bern, University of Bern, Switzerland; ^4^Pediatric Pharmacology and Pharmacogenetics, Hôpital Robert Debré, Paris, France; ^5^Pediatric Emergency Department, Foundation IRCCS Ca' Granda Ospedale Maggiore Policlinico, Milan, Italy; ^6^Pediatric Unit, Università degli Studi di Milano, Foundation IRCCS Ca' Granda Ospedale Maggiore Policlinico, Milan, Italy

## Abstract

Acute nonspecific, or primary, mesenteric lymphadenitis is a self-limiting inflammatory condition affecting the mesenteric lymph nodes, whose presentation mimics appendicitis or intussusception. It typically occurs in children, adolescents, and young adults. White blood count and C-reactive protein are of limited usefulness in distinguishing between patients with and without mesenteric lymphadenitis. Ultrasonography, the mainstay of diagnosis, discloses 3 or more mesenteric lymph nodes with a short-axis diameter of 8 mm or more without any identifiable underlying inflammatory process. Once the diagnosis is established, supportive care including hydration and pain medication is advised. Furthermore, it is crucial to reassure patients and families by explaining the condition and stating that affected patients recover completely without residuals within 2–4 weeks.

## 1. Introduction

Acute nonspecific, or primary, mesenteric lymphadenitis is a self-limiting inflammatory condition affecting the mesenteric lymph nodes [1, 2] (throughout the text, the prefix “nonspecific” is implied when this term is used, unless otherwise stated). Textbooks generally consider this rather common condition no more than a medical curiosity, which is essential exclusively from the standpoint of differential diagnosis with appendicitis and intussusception. Recently performed imaging studies among subjects with suspected appendicitis or intussusception confirm that acute mesenteric lymphadenitis is the most frequent alternative diagnosis [3, 4]. Although liberal use of high-quality imaging studies now better characterizes mesenteric lymphadenitis, its natural history and appropriate management have not been clearly defined. The purpose of this report is to summarize available information (even if limited) and guide practicing physicians faced with this condition.

## 2. History

For a long time, enlarged mesenteric lymph nodes in the young were considered invariably due to tuberculosis. A few years after the First World War, the existence of a mesenteric lymphadenitis, as an independent clinical entity of nontuberculous origin, was recognized [5]. At that time, a definite diagnosis of mesenteric lymphadenitis was very difficult to make before surgery [5]. Unsurprisingly, it was stated that “acute mesenteric lymphadenitis is almost invariably confused with acute appendicitis” and that “there is hardly an abdominal condition for which laparotomy is commonly performed that may not be simulated by diseased mesenteric glands” [6]. Initially, surgical management of mesenteric lymphadenitis with appendectomy was advised by some. Very soon, however, it was recognized that there is no reason to believe that appendectomy affects the disease course, which in any case is self-limiting and from which ultimate recovery seems to be invariable [5, 6]. Many physicians currently consider mesenteric lymphadenitis a nondisease and its symptoms unexplained.

## 3. Definitions: Etiological Hypothesis

Mesenteric adenitis can be divided into two groups: nonspecific (or primary) and secondary. Primary mesenteric adenitis is a lymphadenopathy, mostly right-sided, without an identifiable acute inflammatory process. Secondary mesenteric adenitis is associated with a detectable intraabdominal inflammatory process [7].

## 4. Clinical Presentation

Mesenteric lymphadenitis typically occurs in children, adolescents, and young adults of both sexes, although males might be slightly more frequently affected than females [1–4, 8–10]. Mesenteric lymphadenitis is likely more common than acute appendicitis in the first decade of life [1–4, 8–10]. Acute appendicitis becomes more frequent in the second decade, whilst mesenteric lymphadenitis is distinctly uncommon after the age of 20 years. It often follows or occurs in association with an upper respiratory illness. Relevant symptoms and signs of mesenteric lymphadenitis include the following [1–4, 8–10]:

- Fever ranges between 38.0 and 38.5°C, vomiting, and shifts in stool frequency and consistency are frequently reported.
- Pain is usually severe, but, as a rule, the patient does not appear to be severely prostrated. The character of pain varies from a discomfort to a severe colic. The distribution of pain, like that of appendicitis, is felt both in the periumbilical region and in the right iliac fossa.
- Tenderness is maximal in the right iliac fossa but is often present higher up towards the epigastrium (although this is also a not uncommon site of pain in appendicitis). In our experience, the degree of tenderness is noticeably less in lymphadenitis than in appendicitis and the pressure of the hand will be tolerated by a child suffering from lymphadenitis in a manner not to be found in one with an inflamed appendix. The tenderness is also felt more deeply in lymphadenitis. In the latter, the area of pain tends to shift when the child is moved from side to side (this is in contrast with the more fixed area of tenderness in appendicitis). Rebound tenderness is present also in about one-quarter of the patients [1–4, 8–10] with mesenteric lymphadenitis (probably being due to involvement of the overlying mesentery). True abdominal rigidity is usually absent.

## 5. Laboratory Exams: Imaging Studies

The presentation of mesenteric lymphadenitis may clinically mimic acute appendicitis, intussusception, constipation, inflammatory bowel diseases, Meckel's diverticulum, ovarian torsion, basal pneumonia, Henoch-Schönlein syndrome, and urinary tract infection [1–4, 8–10].

**Figure 1:**
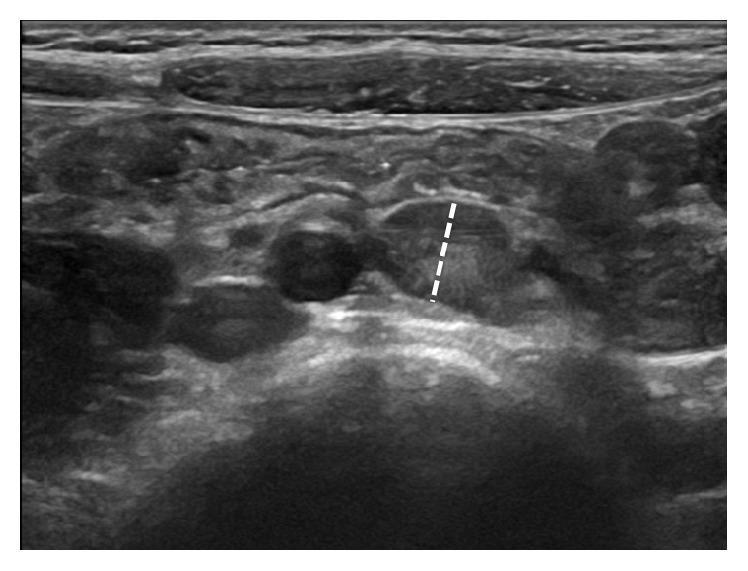
Abdominal ultrasound showing large hypoechoic mesenteric lymph nodes in a 6-year-old girl with acute nonspecific mesenteric lymphadenitis. The largest mesenteric lymph node short-axis diameter (dashed line) measurement was 9 mm.

Like in many cases with acute abdominal pain, white blood cell count and C-reactive protein, a routine part of the diagnostic workup, are often mildly-to-moderately elevated in patients with mesenteric lymphadenitis. Yet, these investigations are of very limited usefulness in distinguishing between patients with and without mesenteric lymphadenitis [3]. Urinalysis may be useful to exclude urinary tract infection. Abdominal ultrasonography is the mainstay of diagnosis. In subjects affected by acute mesenteric lymphadenitis, ultrasonography discloses multiple, enlarged, hypoechoic mesenteric lymph nodes (the absence of a thickened blind-ending tubular structure in the right lower quadrant also suggests the diagnosis of mesenteric lymphadenitis). The radiological definition for mesenteric lymphadenitis suggested more than 20 years ago is a cluster of three or more lymph nodes with short-axis diameter of 5 mm or more in the right lower quadrant and in the para-aortic region without an identifiable acute inflammatory process [11]. More recent data (and our everyday clinical practice) suggest that using a short-axis diameter of 8 mm or more in at least one of the abnormally enlarged lymph nodes (Figure 1) might be a more suitable definition for this condition [8, 12, 13]. Lymph node enlargement is also found in some cases of appendicitis (especially in cases where the appendix is perforated) but generally the nodes are not as numerous nor as large as those visualized in patients with mesenteric lymphadenitis [3]. Malignancies, most frequently non-Hodgkin lymphomas, sometimes have abdominal masses and may result in right lower quadrant tenderness. Concurrent involvement of mesenteric, retroperitoneal, and pelvic lymph nodes is common in these cases.

## 6. Primary versus Secondary Mesenteric Lymphadenitis

Primary or nonspecific mesenteric lymphadenitis has been usually defined as right-sided lymphadenopathy without an identifiable underlying inflammatory cause. In these patients, there are no further imaging abnormalities, except for a slight thickening of the terminal ileum wall and caecum in a minority of cases [14]. On the other side, appendicitis, inflammatory bowel diseases, and, more rarely, systemic chronic inflammatory diseases such as systemic lupus erythematosus, sarcoidosis, and chronic granulomatous disease are causes of secondary mesenteric lymphadenitis (see the following list).

*Causes of Mesenteric Lymphadenopathy other than Acute Nonspecific Mesenteric Lymphadenitis in Children, Adolescents, and Young Adults*

*Chronic (or Subacute) Presentation*

- Inflammatory bowel diseases
- Systemic chronic inflammatory diseases (e.g., systemic lupus erythematosus, and sarcoidosis)
- Malignancy
- HIV infection
- Tuberculosis

*Acute Presentation*

- Appendicitis
- Secondary mesenteric lymphadenitis of infectious origin
- Zoonotic infections: yersiniosis (*Yersinia enterocolitica* or* pseudotuberculosis*) and nontyphoidal* Salmonella* infection
- Enteric fever
- Infectious mononucleosis (Epstein-Barr virus,* Toxoplasma gondii*, and* Bartonella henselae*)

Finally, contrary to common belief, there is no connection between celiac disease and mesenteric lymphadenitis. In most cases of mesenteric lymphadenitis, an underlying viral infectious terminal ileitis is thought to be the cause. Mesenteric lymphadenitis has also been observed in the context of well-defined zoonotic infections such as yersiniosis (caused either by* Yersinia enterocolitica* or by* Yersinia pseudotuberculosis*) and nontyphoidal* Salmonella* infection. We recommend stool testing for these germs exclusively if mesenteric lymphadenitis follows or occurs in association with a diarrheal disease (especially if bloody). Rarely, mesenteric lymphadenitis has also been associated with enteric fever and Epstein-Barr virus,* Toxoplasma gondii,* or* Bartonella henselae* infection. We advise testing for these causes of glandular fever if mesenteric lymphadenitis is associated with findings such as swollen cervical lymph nodes, sore throat, splenomegaly or hepatomegaly, and peripheral blood absolute or atypical lymphocytosis. Finally, mesenteric lymphadenitis has also been observed in HIV patients.

## 7. Management and Outcome

The first objective of management is to quickly identify patients who require surgical intervention and to refer them appropriately. As previously stated, acute mesenteric lymphadenitis is self-limiting: it is assumed but not proven that abdominal pain disappears within 2-3 weeks. Once the diagnosis is definitely established, supportive care including hydration and pain medication with paracetamol or a nonsteroidal anti-inflammatory agent is advised. Even more crucial is explaining the diagnosis in a clear and logical way (the presence of enlarged lymph nodes is often a source of anxiety because of the association with malignancy), reassuring patients and families as necessary and stating that although there is mostly no clear-cut cause and specific cure, affected patients recover without sequelae. In our experience, however, removal of fears and concerns about illness can be difficult to achieve in cases with a causally unclear diagnosis like mesenteric lymphadenitis. This impression is supported by the literature: for instance, concerns often increase after patients with abdominal symptoms are assured that no disease is present [15]. Thus, patients and families should be forewarned that progress can be slow in an effort to minimize frustration when rapid improvement does not occur [8]. It is advantageous to think of the time span for recovery in terms of one to four weeks (and occasionally more). Patients may require extra rest until they recover. Many cases, finally, benefit from regularly scheduled physician appointments, which reduce excessive emergency department visits and avoid expensive and inappropriate interventions. Obviously, further studies are required to better delineate the natural history of mesenteric lymphadenitis.
